# Emergency Coronary Artery Bypass Graft Surgery for Iatrogenic Left Main Coronary Artery Dissection

**Published:** 2015-10-27

**Authors:** Masoud Tarbiat, Gholamreza Safarpoor

**Affiliations:** Ekbatan Heart Hospital, Hamedan University of Medical Sciences, Hamedan, Iran.

**Keywords:** *Coronary vessels*, *Dissection*, *Coronary artery bypass*

## Abstract

Iatrogenic coronary artery dissection during coronary angiography with or without rupture is a rare but feared complication. We herein report a case of iatrogenic left main coronary artery dissection in a 49-year-old female. Admitted to our hospital with a recent history of severe hypotension, she develpled apnea during angiography. She was intubated and resuscitated with an Epinephrine infusion in the Cath-Lab. The diagnosis was iatrogenic left main coronary artery dissection based on angiography. Immediately, the patient was transferred to the operating room in a lethargic state with an Epinephrine infusion and prepared for emergency coronary artery bypass graft surgery. In the ICU, she was completely alert with no hemodynamic complications and finally was discharged in a good overall condition. At 18 months' follow-up, the patient was in a stable situation with good daily function.

## Introduction

Iatrogenic left main coronary artery (LMCA) dissection, induced by catheter insertion, is one of the most feared complications, albeit with a reported incidence rate of less than 0.1%.^1^ Its occurrence can have devastating consequences, if not promptly treated with immediate revascularization. The LMCA dissection often leads to abrupt vessel closure and cessation of the blood flow toward a large portion of the myocardium, resulting in acute pump failure and hemodynamic collapse.^2^ Conservative treatment may be adequate for limited dissections, but extensive dissections with pericardial extravasation require immediate treatment.^3^ Both emergency coronary artery bypass graft surgery (CABG) and use of covered stents consitute effective in treatment for the LMCA dissection. 

Here we present a case of iatrogenic LMCA dissection following angiography, which was treated successfully with emergency CABG.

## Case Report

**Figure 1:**
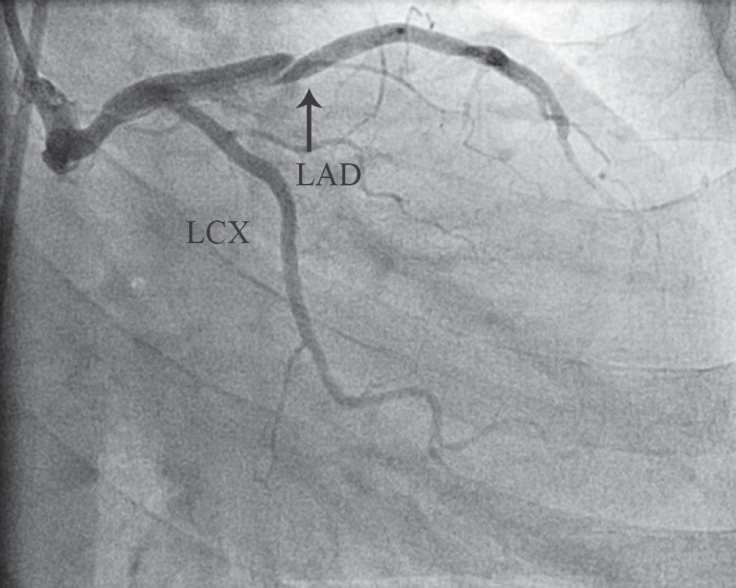
left anterior descending (LAD) artery dissection (arrow) in a coronary angiogram (right anterior oblique view)

**Figure 2:**
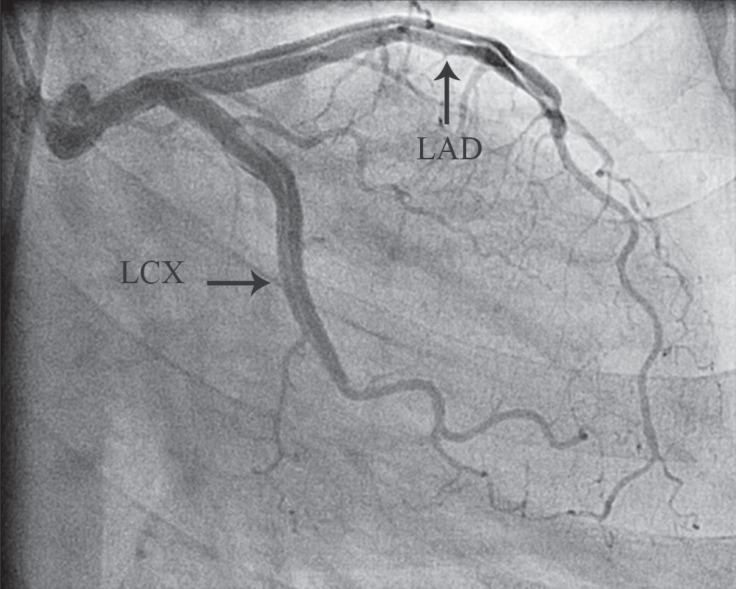
Left main coronary artery dissection, extending to the left anterior descending (LAD) and left circumflex (LCX) arteries (Type B) in the right anterior oblique view (arrows)

A 49-year-old obese opium-addicted female patient with a history of mild hypertension, asthma, and chest pain of 3 months' duration was admitted for coronary angiography in July 2012. In the Cath-Lab during angiography, she developed severe hypotension and apnea. Therefore, she was intubated and resuscitated with an Epinephrine infusion without delay. The intubated patient was subsequently referred to our hospital for emergency coronary artery bypass graft surgery (CABG). Coronary angiography reported the dissection of the left main coronary artery (LMCA) (Type B), extending to the immediate distal portion of the left anterior descending artery (LAD) and the mid portion of the left circumflex artery (LCX) (Figures 1 and 2). Echocardiography revealed ejection fraction of 55%.

On admission, the patient was still intubated and was in a stable hemodynamic state with an Epinephrine infusion. Without dealy, she was transported to the operating room in a lethargic state and was prepared for the induction of anesthesia. In the operating room, the patient was monitored via standard electrocardiography and pulse oximeter. The veins on both arms were cannulated with 16-G catheters under local anesthesia with Lidocaine (1%). The catheter was inserted in the left radial artery to monitor blood pressure. The patient was induced with Sufentanil (50 μg), Etomidate (14 mg), and Cisatracurium (14 mg). Anesthesia was maintained using an infusion of Propofol (50-75 μg/kg/min), Sufentanil (2 μg/kg/h), Cisatracurium (2 μg/kg/min), and Dexamethasone (8 mg). Additionally, a vitamin C infusion (500 mg) was administered. Subsequently, a tri-lumen catheter was inserted in the right subclavian vein.

**Figure 3:**
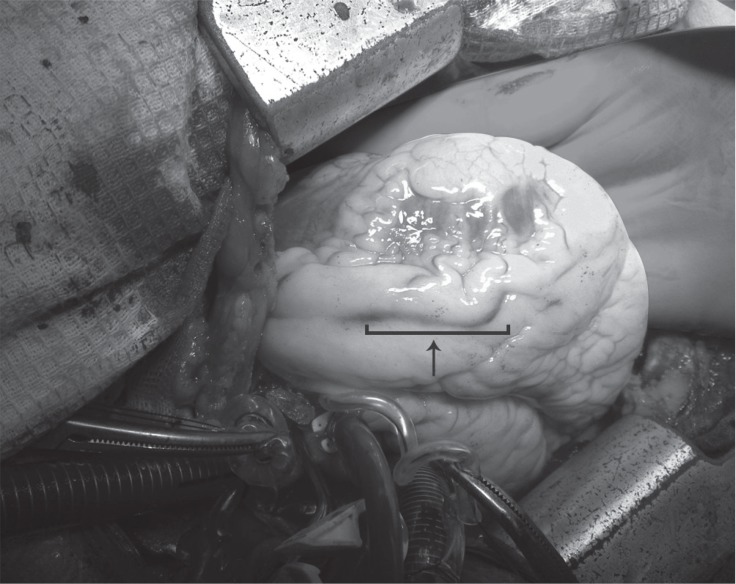
Intraoperative view of the left anterior descending (LAD) artery dissection (arrow)

Under general anesthesia, median sternotomy was performed, followed by the harvesting of the left internal mammary artery (LIMA) and the greater saphenous vein from left lower extremity. Operative findings confirmed the dissection and that it extended up to the immediate distal portion of the LAD and the mid portion of the LCX (Figure 3). Systemic Heparinization (300 IU/kg) was commenced, and aortic and atrial purse sutures were applied with 2/0 Prolene. Thereafter, the ascending aorta and the right atrium were cannulated (two-stage cannulae), cardio-pulmonary bypass was established, and systemic cooling was continued to reach 33 ºC. Next, aortic cross-clamping was performed, followed by the infusion of an antegrade cold cadioplegic solution into the aortic root, achieving complete cardiac arrest (cold blood). Using 8/0 Prolene, the LIMA and saphenous vein graft (SVG) were anastomosed to the distal portions of the LAD and the LCX, respectively. All the grafts were positioned and checked successfully. Return of normal sinus rhythm was uncomplicated following rewarming.Weaning from cardiopulmonary bypass was performed with a low-dose Epinephrine infusion (0.05 μg/kg/min).

The operation was uneventful, and the patient was transferred to the Open Heart Intensive Care Unit, where she was extubated after 6 hours in complete alertness and without any hemodynamic complications. She was finally discharged home in a good overall condition. On the last follow-up visit (January 2014), she was in a stable situation with good daily function.

## Discussion

Coronary angiography has become a routine diagnostic procedure in current clinical practice.^3^ Complications, although uncommon, still occur during coronary angiography and can prove life-threatening in some instances. Catheter-induced dissection of a coronary artery is a rare but well-recognized complication of coronary angiography with a high mortality rate if left untreated.^3^^, ^^4^ The exact mechanism of iatrogenic coronary artery dissection is not clear. It has been related to vigorous hand-injection of contrast medium, subintimal passage of the guide-wire, or inappropriate handling of the guide-wire catheter.^5^ In all instances, mechanical straining and shearing forces during coronary angiography result in increased wall stress.^3^ Most dissections are focal and limmited to the site of the injury; they rarely extend and propagate from the original site of the injury, affecting much more of the artery. The term "*spiral dissection*" is often used to discribe a dissection that spreads from the site of the original intimal tear and extends the length of the artery. Usually, dissections propagate in the direction of the arterial blood flow. Thus, they typically extend distal to the site of the initial injury; it is very unusual for a dissection to propagate proximally. The development of an aortic dissection complicating a coronary dissection is very rare.^6^

The National Heart Lung and Blood Institute (NHLBI) classification system for coronary artery dissection types are as follows: Type A dissections represent minor radiolucent areas within the coronary lumen during contrast injection with little or no persistence of contrast after the dye has cleared. Type B dissections are parallel tracts or a double lumen separated by a radiolucent area during contrast injection, with minimal or no persistence after dye clearance. Type C dissections appear as contrast outside the coronary lumen (extraluminal cap) with the persistence of contrast after dye has cleared from the lumen. Type D dissections represent spiral (barber shop pole) luminal filling defects, frequently with excessive contrast staining of the dissected false lumen. Type E dissections appear as new, persistent filling defects within the coronary lumen. And, Type F dissections represent those that lead to the total occlusion of the coronary lumen without distal antegrade flow. Types A and B are generally clinically benign, whereas Types C through F portend significant morbidity and mortality if untreated.^7^ In rare cases, a coronary artery dissection may propagate retrogradely and involve the ascending aorta.^8^

Iatrogenic LMCA dissection results from mechanical injury to the arterial wall during catheter manipulation or the passage or deployment of an interventional device. Extensive catheter manipulation, catheter type (e.g. Amplatz catheter and small Judkins catheter resulting in deep LMCA intubation), stiffer and less manageable guide-wires (e.g. pressure wire), unusual LMCA anatomy or location, operator's experience, and presence of the LMCA atherosclerosis have all been associated with an increased risk of dissection.^2^^,^ 9

Once dissection occurs, the clinical picture differs, depending on the remaining antegrade coronary flow, and can range from an asymptomatic patient to a patient in refractory cardiogenic shock whose LMCA is "*amputated*". Nevertheless, even in case of hemodynamic stability, rapid deterioration may occur shortly afterward because of abrupt flow compromise due to progressive dissection or superimposed thrombus formation, which is why it seems imperative to always envisage a "*revascularization plan*" via percutaneous coronary intervention (PCI) or CABG.^2^

In case of acute LMCA dissection, the technique of surgery and myocardial protection is extremely important. Retrograde coronary perfusion should be strongly considered in these cases along with controlled warm reperfusion. In our patient, however, after the infusion of an antegrade cold cardioplegic solution into the aortic root, complete cardiac arrest occurred. Therefore, it was not necessary to use retrograde coronary perfusion. Although we did not employ this method, it merits consideration it in this section. Awadalla et al.^1^ reported iatrogenic LMCA dissection with an incidence of less than 0.1%.^1^ Eshtehardi et al.^10^ stated that the overall incidence of iatrogenic LMCA dissection during their study period was 0.07% and almost twice as common with PCI than coronary angiography. From their 38 patients, 1 (3%) patient died before any therapeutic attempt was performed, 6 (16%) patients were treated conservatively, and 31 (82%) patients underwent stent implantation and/or CABG. In their study, the in-hospital outcome was favorable irrespective of the therapeutic strategy. During their 5-year follow-up, the authors reported that among the 31 patients who underwent revascularization treatment by stenting or CABG, 1 patient died in each group from a cardiac cause, denoting that there were no significant differences between the different revascularization treatment strategies.^10^ In contrast to Demetrios Antoniades et al.,^3^ who believe emergency CABG is a time-consuming procedure that entails the risk of irreversible myocardial damage, our case showed that in some situations, it can prove a life-saving method without any complications. 

## Conclusion

Iatrogenic LMCA dissection is a rare complication of coronary angiography procedures with favorable early and long-term outcomes when recognized timely and managed properly. Furthermore, emergency CABG is still an acceptable option for the treatment of this complication.

## Notes:

***This paper should be cited as:*** Tarbiat M, Safarpoor G. Emergency Coronary Artery Bypass Graft Surgery for Iatrogenic Left Main Coronary Artery Dissection. J Teh Univ Heart Ctr 2015;10(4):215-218.

## References

[1] Awadalla H, Sabet S, El Sebaie A, Rosales O, Smalling R (2005). Catheter-induced left main dissection incidence, predisposition and therapeutic strategies experience from two sides of the hemisphere. J Invasive Cardiol.

[2] Onsea K, Kayaert P, Desmet W, Dubois CL (2011). Iatrogenic left main coronary artery dissection. Neth Heart J.

[3] Antoniades D, Apostolakis S, Tzoras S, Lazaridis K (2009). Iatrogenic right coronary artery dissection distal to a total occlusion: a case report. Cases J.

[4] Garcia-Robles JA, Garcia E, Rico M, Esteban E, Perez de Prado A, Delcan JL (1993). Emergency coronary stenting for acute occlusive dissection of the left main coronary artery. Cathet Cardiovasc Diagn.

[5] El-Jack SS, Pornratanarangsi S, Webster MW (2006). Images in cardiology. Covering your mistakes: PTFE covered stents in iatrogenic coronary dissection. Heart.

[6] Micheal R, Micheal R (2011). Coronary dissection involving the aortic root. Cases in Interventional Cardiology.

[7] Bittl JA, Ryan TJ Jr, Keaney JF Jr, Tcheng JE, Ellis SG, Isner JM, Sanborn TA (1993). Coronary artery perforation during excimer laser coronary angioplasty The percutaneous Excimer Laser Coronary Angioplasty Registry. J Am Coll Cardiol.

[8] Goldstein JA, Casserly IP, Katsiyiannis WT, Lasala JM, Taniuchi M (2003). Aortocoronary dissection complicating a percutaneous coronary intervention. J Invasive Cardiol.

[9] Kovac JD, Spyt TJ, Firmin RK, Verma PK, de Bono DP (1999). Chronic haemoptysis as delayed complication of ventricular aneurysmectomy. Int J Cardiol.

[10] Eshtehardi P, Adorjan P, Togni M, Tevaearai H, Vogel R, Seiler C, Meier B, Windecker S, Carrel T, Wenaweser P, Cook S (2010). Iatrogenic left main coronary artery dissection: incidence, classification, management, and long-term follow-up. Am Heart J.

